# Quantitative MALDI-TOF Mass Spectrometry of Star-Shaped Polylactides Based on Chromatographic Hyphenation

**DOI:** 10.1021/jasms.4c00491

**Published:** 2025-01-30

**Authors:** Jana Falkenhagen, Mete-Sungur Dalgic, Steffen M. Weidner

**Affiliations:** Federal Institute for Materials Research and Testing (BAM), Richard-Willstätter-Strasse 11, D-12489 Berlin, Germany

## Abstract

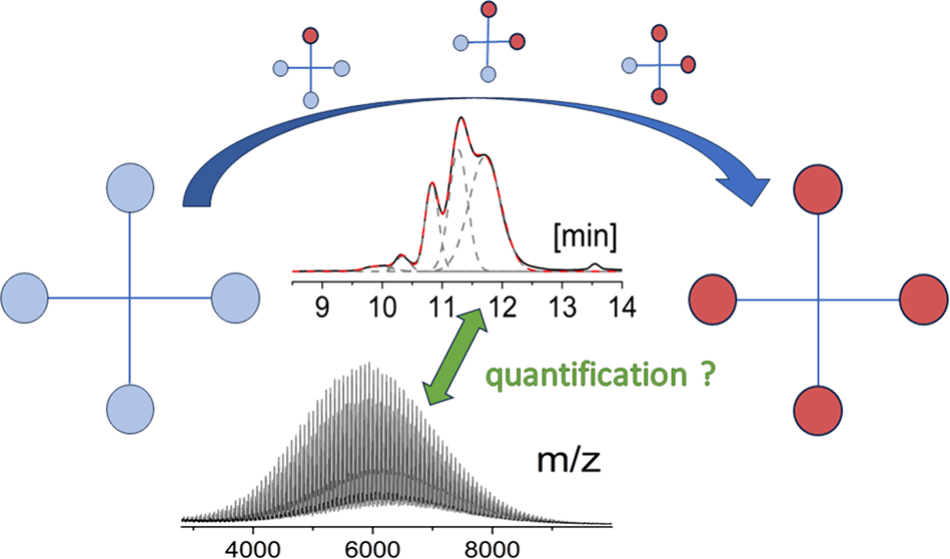

The end groups of three- and four-arm star-shaped polylactides (PLA) with trimethylolpropane and pentaerythritol core structures were functionalized with acetic acid. Reaction products with different degrees of functionalization were analyzed by matrix-assisted laser desorption/ionization time-of-flight (MALDI-TOF) mass spectrometry. Additional gradient elution liquid adsorption chromatography (GELAC) measurements were performed to determine the degree of functionalization. This technique enabled clear separation and sufficient quantification of the formed species. These chromatographic data could be used inversely to quantify mass spectrometric results, which are usually biased by the unknown ionization probabilities of different polymer end group structures. Our results showed that, in this particular case, the peak intensity in the MALDI-TOF mass spectra can be used to semiquantitatively determine the degree of functionalization in incompletely functionalized multiarm PLA.

## Introduction

MALDI-TOF mass spectrometry represents a technique for the simultaneous determination of structures, end groups, and molar masses of synthetic polymers. However, this versatility is overshadowed by the lack of quantitative results. Due to the principle of MALDI, which is based on a desorption step accompanied by gas-phase ionization,^1^ mass spectrometric data must always be considered carefully.

Ideally, the structure of an unknown polymer can be deduced from the peak-to-peak distance in the MALDI-TOF mass spectrum. However, in a polymer blend consisting of different polymers or in a copolymer build of two or more polymers, preferential ionization of one polymer structure or discrimination of another polymer can result in a significant loss of information about a major part of the total sample.^2^ Moreover, the determination of the molecular mass of a polymer is affected by its dispersity, which should typically be around 1.2 to give reasonable results. MALDI-TOF mass spectra of blends of polymers with identical structures but different molar masses typically show a dramatic decrease in intensities in the higher mass range. Various explanations have been given in the literature, to interpret such mass discrimination effects.^3^

Among other issues, such as cation interference^4^ and cation selectivities of different polymers^5^ or the way the sample spots were prepared (e.g., solvent-based, solvent-free, electro spray, etc.),^6^ the question of whether the structure of the polymer end group affects ionization is still not sufficiently answered. Controversial statements about this can be found in the literature. Puglisi et al. found that end group ionization efficiency appears to be the most important parameter in determining the relative intensity of peaks in the MALDI-TOF mass spectra of nylon-6 and poly(butylene terephthalate) blends with different varying end groups. They postulated that understanding the mechanisms of ionization efficiency is essential for quantitative applications of MALDI-TOF MS.^7^ In another paper, polylactides bearing different types of end groups (e.g., cationic, easy ionizable, and neutral) were investigated. There, it was found that the extent of signal intensity does not depend on the ability of the cation (Li^+^, Na^+^, K^+^) to undergo ionization but rather depends on the affinity of the given end group to a proton or metal cation.^8^ On the other hand, when investigating blends of linear polylactides with different end groups, Weidner and Kricheldorf found that the polarity of the end groups did not significantly affect the ionization efficiency, which clearly supports their assumption that ionization in MALDI is unlikely to be affected by the structure of the end groups (in polylactides).^9^ These contradictory data allow the following conclusion to be drawn. Since ionization in polymer MALDI typically occurs by cation attachment to a polymer chain, the location of this process obviously depends on both the structure of the end groups and the structure of the polymer. This means that polar polymers (e.g., with different heteroatoms) will be ionized by statistical (random) ion adduct formation at any monomer unit of the polymer chain, mostly unaffected by a single end group, whereas nonpolar polymers with more polar end groups provide a very specific site where attachment must necessarily occur. Two illustrative examples will be presented. Wallace and colleagues employed a covalent bonding technique to attach an organic cation to the end of a polyolefin chain.^10^ Kona et al. modified double bonds of polydienes by epoxidation, thereby introducing polar centers within the polymer chain.^11^ Both techniques resulted in the formation of necessary sites for ionization at different places of the polymer chain and led to the creation of intact gas-phase ions of polymers that usually were impossible to ionize.

Our approach, the stepwise functionalization of end groups of a multiarm polymer, offers for the first time the possibility to systematically study the contribution of a particular end group to ionization in MALDI-TOF mass spectrometry. Since the complete functionalization results in a moderate shift of the molar masses of the stars, the observed effects can be exclusively attributed to the modification of the end groups.

**Scheme 1:**
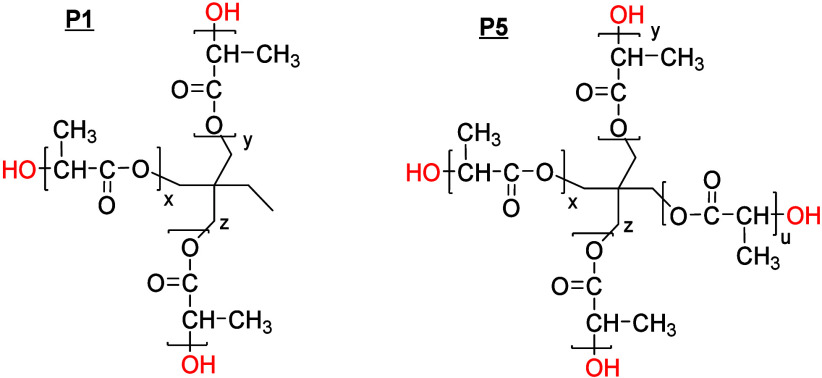
Structure of Star-Shaped Polylactides Used for Functionalization

Another objective was to verify the limits of detection of MALDI-TOF MS for determining minor components of a complex polymer mixture. For our investigation, three- and four-arm star-shaped polylactides (Scheme 1) with trimethylolpropane and pentaerythritol core structures were synthesized and successively functionalized with acetic acid anhydride. Since sufficient chromatographic resolution was a prerequisite for our study, polymer stars with an even larger number of arms (e.g., eight-arm structure by starting from a tripentaerythritol core or even a dendrimer structure) could not be investigated so far.

For the chromatographic separation, we used gradient elution liquid adsorption chromatography (GELAC) in combination with an evaporative light scattering detector (ELSD). The most important feature of this combination is that the baseline cannot drift with changing the solvent composition of the gradient. This technique was first applied to polymers by Schoenmakers et al. and Biedermann and Grob^12^ and is meanwhile a unique tool in 2D chromatographic separations where large quantities of solvents are collected in the first dimension and then transferred to the second dimension.^13^ However, it has been shown that the use of a gradient as a mobile phase can cause quantification problems. There have been some reports in the literature that the response factors for analytes were not constant across the gradient.^14,15^ Reasons for this could be that the organic content of the mobile phase can change both the transport efficiency of the nebulizer and the particle size distribution.^15^ However, the development of this detection technique over the past 15 years allows it to be used for quantification under certain conditions. First, the chromatographic experiments were carried out at constant temperature. Second, the molar masses of all analytes were on the same order of magnitude. Third, the polymers were of virtually the same chemical nature; i.e., they consisted of the same repeating units. Finally, possible inflictions caused by the use of solvent gradients were minimized since the elution took place in a narrow time range of 3.5 min, during which the composition of the mobile phase (i.e., the amount of CHCl_3_) changed linearly from 58% to 76%.

Therefore, assuming nearly quantitative data from GELAC, the peak intensities of both methods can be correlated, thus allowing a quantification of the MALDI data.

## Experimental Section

### Materials

l-(−)-lactide (LA) and *trans*-2-[3-(4-*tert*-butylphenyl)-2-methyl-2-propenylidene] malononitrile (DCTB) were supplied by Tokyo Chemical Industry Co. Ltd. (Tokyo, Japan). Tin(II) 2-ethylhexanoate (SnOct_2_), pyridine, acetic anhydride, potassium trifluoroacetate (KTFA), and 1,1,1-tris(hydroxymethyl)propane (TMP) were obtained from Sigma-Aldrich (Taufkirchen, Germany). Toluene, *n*-hexane (both HPLC grade), and pentaerythritol (PENT) were purchased from Carl Roth (Karlsruhe, Germany). 1,4-Dioxane was obtained from Th. Geyer (Renningen, Germany).

### Synthesis of the Three-Arm PLA Star (TMP–OH) **P1**

l-lactide (40 mmol) and trimethylolpropane (1 mmol) were weighed in a 50 mL Erlenmeyer flask. After adding a solution of SnOct_2_ (0.4 M) in toluene (2 mL), the reaction vessel was closed with a glass stopper and a clamp and immersed into an oil bath, which was heated to 120 °C. After 40 min, the reaction was aborted, and the flask was taken out of the oil bath. The solid product was mechanically removed from the reaction vessel using a spatula.

### Synthesis of the Four-Arm PLA Star (PENT–OH) **P5**

l-lactide (40 mmol) and pentaerythritol (1 mmol) were weighed in a 50 mL Erlenmeyer flask. After adding a solution of SnOct_2_ (0.4 M) in toluene (2 mL), the reaction vessel was closed with a glass stopper and a clamp and immersed into an oil bath, which was heated to 120 °C. After 30 min, the reaction was aborted, and the flask was taken out of the oil bath. When the reaction mixture was solidified, the product was removed from the reaction vessel using a spatula.

### Acetylation of **P1**

Different ratios of **P1** and acetic acid anhydride/pyridine were heated in a mixture of toluene and 1,4 dioxane. First experiments using 500 mg of **P1** (112 μmol), 20 (60) μL of acetic anhydride, and 12.5 (20) μL of pyridine resulted in incomplete functionalization of OH-end groups (experiments TMP-PLA-Ac_1 and TMP-PLA-Ac_2) as proven by MALDI-TOF MS data. For a complete acetylation (TMP-PLA-Ac_3), the amount of acetic anhydride/pyridine was significantly increased. 100 mg of **P1** (22 μmol) and acetic anhydride (1 mL) and pyridine (300 μL) were heated in a larger volume of a refluxing mixture of toluene (8 mL) and 1,4-dioxane (8 mL). After 4 days, the reaction mixture was cooled, and the functionalized PLA precipitated into 150 mL of *n*-hexane.

### Acetylation of **P5**

The acetylation of the four-arm PLA (**P5**) proceeded similarly. First, lower acetic anhydride/pyridine amounts were used (experiment PENT–PLA-Ac_1), which again resulted in an incomplete degree of functionalization. For example, 30 mg of **P5** (6 μmol) and 45 μL of acetic anhydride and 20 μL of pyridine were heated in a refluxing mixture of toluene (4 mL) and 1,4-dioxane (4 mL). After 6 h, the reaction mixture was cooled, and the product was precipitated into 100 mL of *n*-hexane. For a complete functionalization (experiment PENT–PLA-Ac_2), 80 mg of **P5** (15 μmol) and acetic anhydride (1.2 mL) and pyridine (400 μL) were used and heated in a refluxing mixture of toluene (8 mL) and 1,4-dioxane (8 mL). After 3 days, the reaction mixture was cooled, and the functionalized PLA was precipitated into 100 mL of *n*-hexane.

### Measurements

Size exclusion chromatography (SEC) was carried out on a SECurity GPC system (PSS GmbH, now Agilent) equipped with a refractive index detector (40 °C), three Phenogel columns (1 × 10^5^ Å; 1 × 10^3^ Å; 1 × 100 Å, each 5 μm; 7.8 × 300 mm), and a security guard cartridge (5 μm, 4 × 3 mm). The column temperature was 40 °C. Samples were dissolved in trichloromethane (CHCl_3_, HPLC grade, 2–3 mg mL^–1^). A 100 μL portion of each sample solution was injected at a flow rate of 1 mL min^–1^. Calibration was done using polystyrene standards ranging from 370 to 1.2 × 10^7^ g mol^–1^, and additionally, with poly lactide standards 1550 to 1.0 × 10^5^ g mol^–1^. For calibration and data evaluation, PSS WinGPC UniChrom was used.

For gradient elution liquid adsorption chromatography (GELAC), a system equipped with a Discovery Cyano column (Merck, SUPELCO, 5 μm particle size, 120 Å pore size, 250 mm × 4.6 mm) was used. The autosampler and column were operated at 25 °C. The experiments were performed with a mobile phase system of water and CHCl_3_. While the gradient was running, the concentration of CHCl_3_ increased from 10 to 90% within 15 min. The samples were dissolved in a solvent mixture of 70/30 (v/v, CHCl_3_/water). A volume of 20 μL of each sample with a concentration of approximately 1.5 mg mL^–1^ was injected. For detection, an evaporative light scattering detector (ELSD, 1290 Infinity II, Agilent), operating at an 80 °C evaporation temperature and 40 °C nebulizer temperature, was used. Peak fitting and data handling were performed using Origin2023 software (OriginLab, USA).

For the MALDI-TOF MS experiments, an Autoflex maX mass spectrometer (Bruker Daltonik GmbH, Bremen, Germany) with a 355 nm Nd:YAG laser operating at 2000 Hz was used. For sample preparation, 50 μL of DCTB (TCI, Japan) solution (20 mg mL^–1^ in chloroform) was premixed with 5 μL of a KTFA (Sigma-Aldrich) solution (5 mg mL^–1^ in THF) and 20 μL of PLA solutions (1 mmol L^–1^ in chloroform). Finally, 1 μL of this solution was dropped onto a stainless steel target. For all experiments, the instrumental parameters (laser power, delay time, voltages, etc.) were kept constant. Each spectrum was recorded in linear mode by accumulating 8000 laser shots from four randomly selected positions on the sample spot. Several spots (at least three) were measured for better reproducibility. Spectra were evaluated after baseline subtraction.

## Results and Discussion

**Scheme 2:**
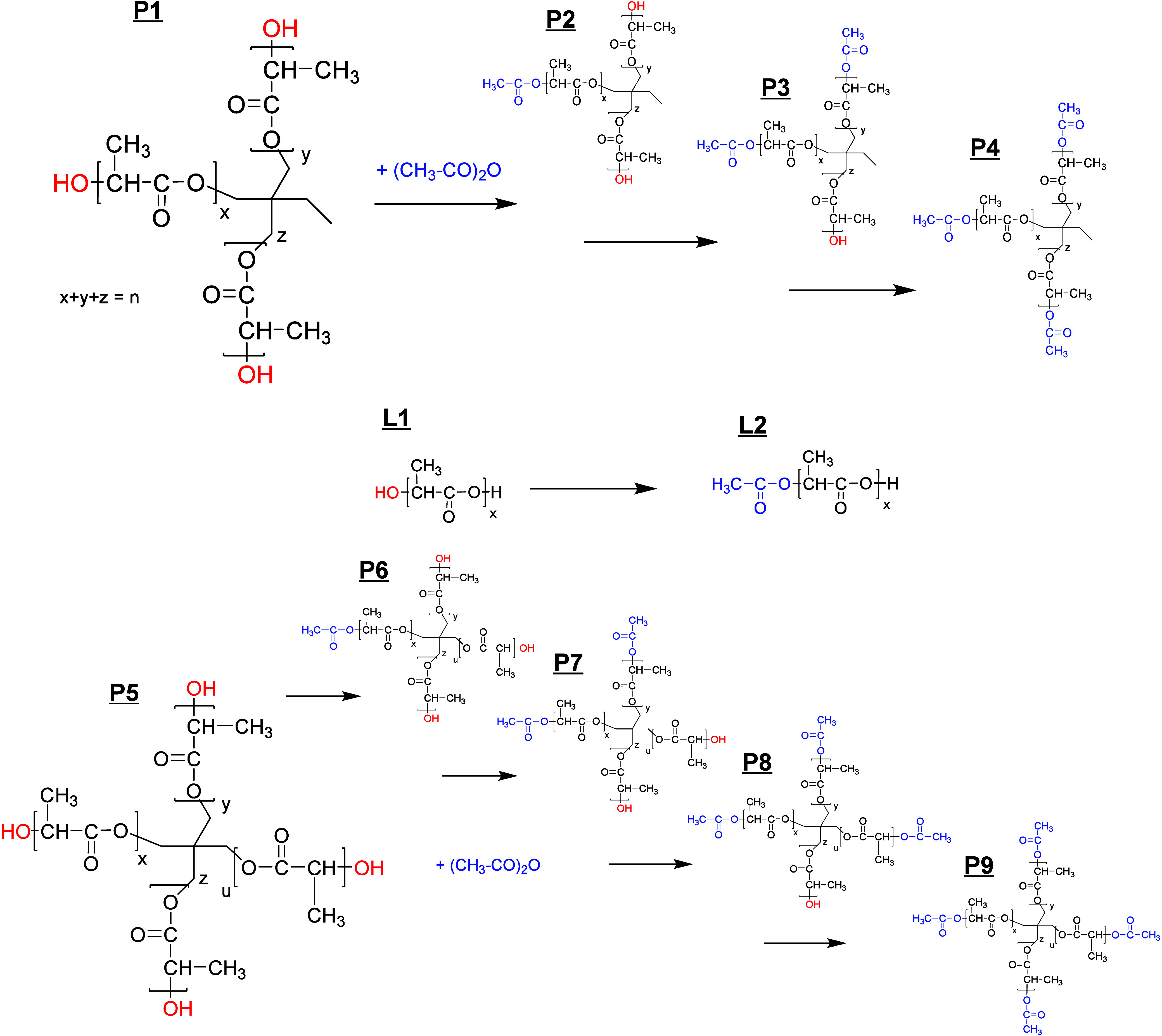
Nomenclature of Reaction Products of Three-Arm PLAs (**P2**–**P4**), Four-Arm PLAs (**P6**–**P9**), and Linear Side Products (**L1**, **L2**)

In a first set of experiments, a three-arm polylactide with three hydroxy end groups (**P1**) and an average molar mass of 5000 g mol^–1^ was modified with acetic acid anhydride. The nomenclature of the reaction products is shown in Scheme 2. The molar masses of the precursors (**P1**, **P5**) and the reaction products are listed in Table S1 (Supporting Information).

**Figure 1:**
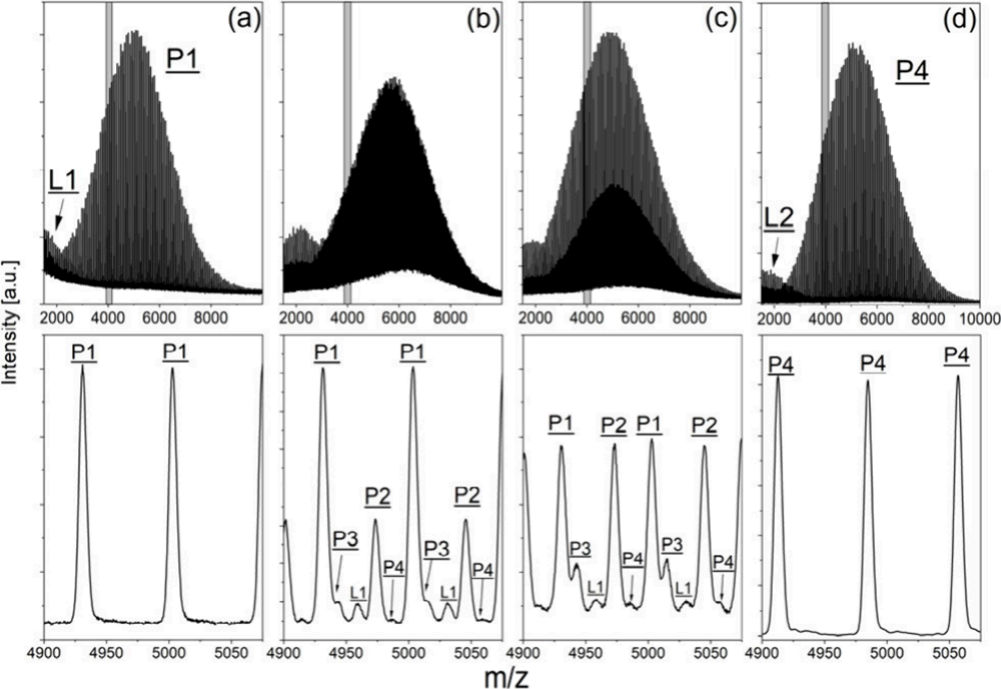
MALDI mass spectra of the original three-arm PLA (**P1**, a) and of three functionalization experiments (b, TMP-PLA-Ac_1; c, TMP-PLA-Ac_2; and d, TMP-PLA-Ac_3). Gray regions are expanded below, showing the change of peak pattern indicating different degrees of functionalization.

As shown in this table, three experiments (TMP-PLA-Ac_1, TMP-PLA-Ac_2, and TMP-PLA-Ac_3) were performed using **P1**, resulting in mixtures of products with different degrees of functionalization (**P2**, **P3**, and **P4** along with the unmodified precursor **P1**, Figure 1). In contrast to the commonly applied SEC calibration with polystyrene standards, which shows a deviation of more than 100% from the MALDI data, the calibration with lactide polymer standards resulted in excellent agreement with the MS data. A similar trend was also found for the four-arm stars (lower part of Table S1), whose functionalization will be discussed later.

**Figure 2:**
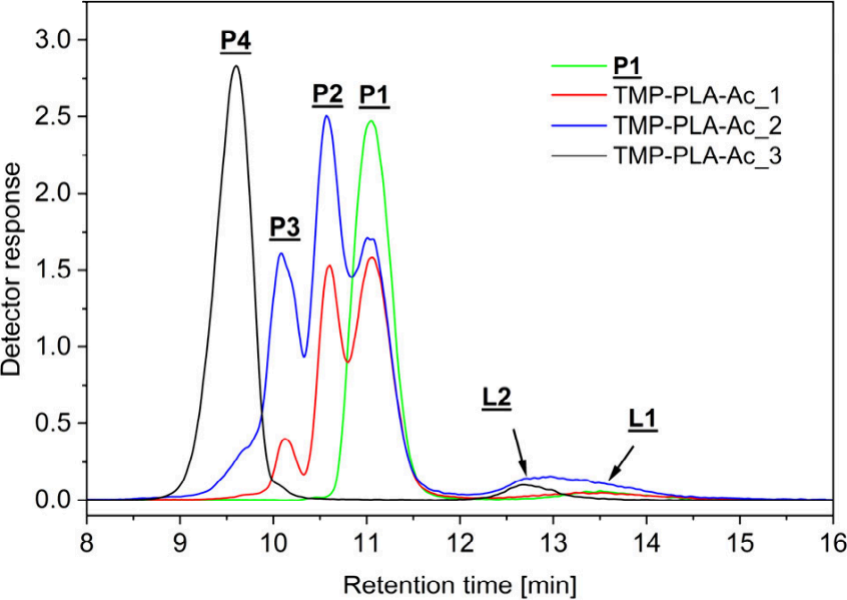
Overlay of GELAC elution curves of the original three-arm TMP-PLA (**P1**) and after functionalization (TMP-PLA-Ac_1, TMP-PLA-Ac_2, and TMP-PLA-Ac_3). The region between 12 and 15 min shows lower mass linear PLA (**L1**, **L2**).

**Figure 3:**
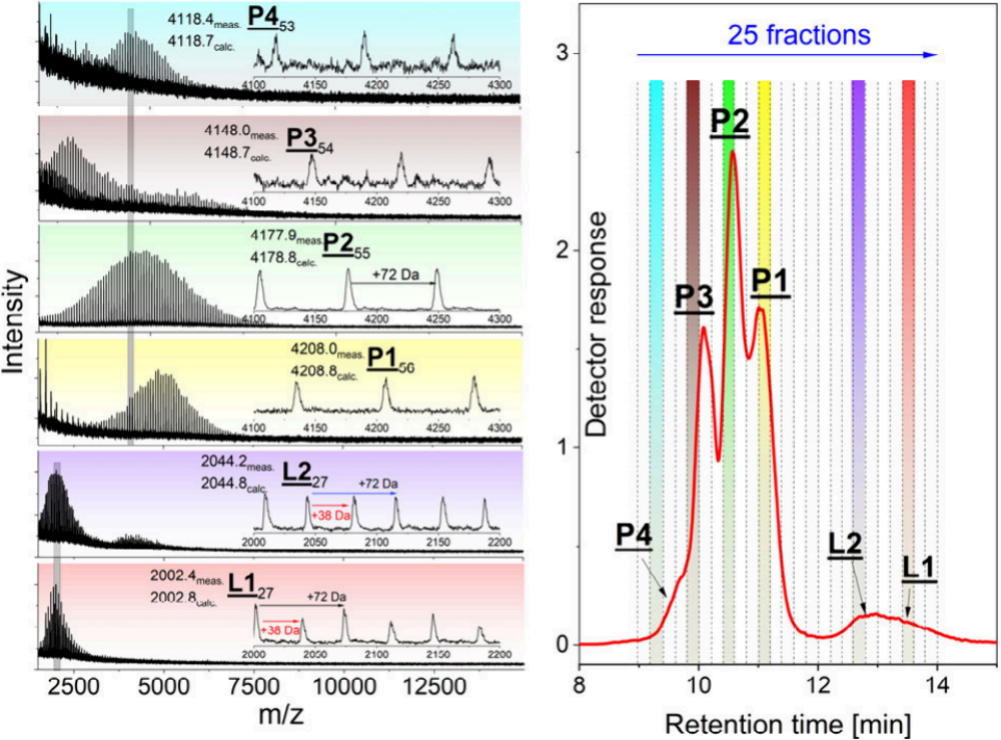
MALDI-TOF mass spectra of characteristic fractions from the GELAC run of sample TMP-PLA-Ac_2 showing various end group functionalities (for assignment, see Scheme 2).

The resulting MALDI-TOF mass spectra are shown in Figure 1. The enlargement of the MS spectra (Figure 1, bottom) shows an increasing complexity, especially in the spectra of TMP-PLA-Ac_1 and TMP-PLA-Ac_2, due to the appearance of new peak series, which were characteristic of three-arm stars with different degrees of functionalization. As shown in Figure 1d, complete functionalization could be achieved by the synthesis route chosen. Here, in the main region of the spectra, only peaks characterizing a polymer star (**P4**) with fully acetylated end groups were visible. However, a small additional peak distribution was found in the lower mass region of all of the spectra. These peaks correspond to shorter linear polymer chains **L1** and, after end group modification, **L2**. These species were apparently formed in a side reaction during the synthesis of the three-arm star (**P1**) and were modified by acetylation, similar to the stars. Since both their elution in the GELAC and their mass peaks in the MALDI spectra are separated from their star-shaped counterparts, they were not part of the intensity comparison discussion later. To obtain more quantitative data on the degree of functionalization, these four samples were then analyzed by GELAC. Their superimposed chromatograms are shown in Figure 2. The curves obtained clearly show a trend from the original three-arm PLA (**P1**) to the fully modified **P4**, as evidenced by the appearance of three additional peaks with a shift in retention time from 11.1 to 9.5 min. An additional region of small but very broad peaks can be seen between 12 and 14.5 min. This is thought to be the elution region of the smaller linear polymers. To confirm our assumptions, manual fractionation of the GELAC runs of incompletely (TMP-PLA-Ac_2) and completely (TMP-PLA-Ac_3) functionalized three-arm PLA stars was performed. The results are shown in Figure 3 (TMP-PLA-Ac_2) and Figure S1 (Supporting Information; TMP-PLA-Ac_3).

Due to the chosen polarity of the separation columns, the interaction of acetylated end groups with the stationary phase was supposed to be less intense than those of OH end groups. Therefore, the higher the degree of functionalization was, the more their elution should be shifted toward lower retention times. This is clearly confirmed by Figure 3. The MALDI-TOF mass spectra of selected fractions shown in Figure 1, taken from incompletely functionalized sample TMP-PLA-Ac_2, clearly confirmed this assumption and showed the existence of all possible end group structures (**P1**–**P4**). As indicated by the very small shoulder at 9.6 min (Figure 2, blue line), only a minor part of the reaction products revealed complete acetylation (**P4**). A major part of the sample still consisted of stars with singly (**P2**) and doubly functionalized (**P3**) end groups beside a considerable amount of the original compound (**P1**). In addition, some interesting details were found in the spectra of the linear side products (**L2** and **L1**). Besides the expected peak-to-peak differences of 72 Da characterizing the PLA repeat units, additional peak series differing by +38 Da were found. This could be easily explained by the additional formation of potassium carboxylate end groups since potassium trifluoroacetate was used as a dopant. A similar behavior has recently been reported in the water-initiated polymerization of l-lactide.^16^ In contrast to that, the results of the fractionation of TMP-PLA-Ac_3 (Figure S1) showed that the acetylation of the hydroxyl end groups of the three-arm star was almost complete. Only a small shoulder at 10 min of retention time indicated the presence of a minor amount of incompletely functionalized PLA (**P3**). Its MALDI mass spectrum also showed an additional series of peaks with a difference of +38 Da indicating the formation of potassium carboxylate at the nonfunctionalized arm.

**Figure 4:**
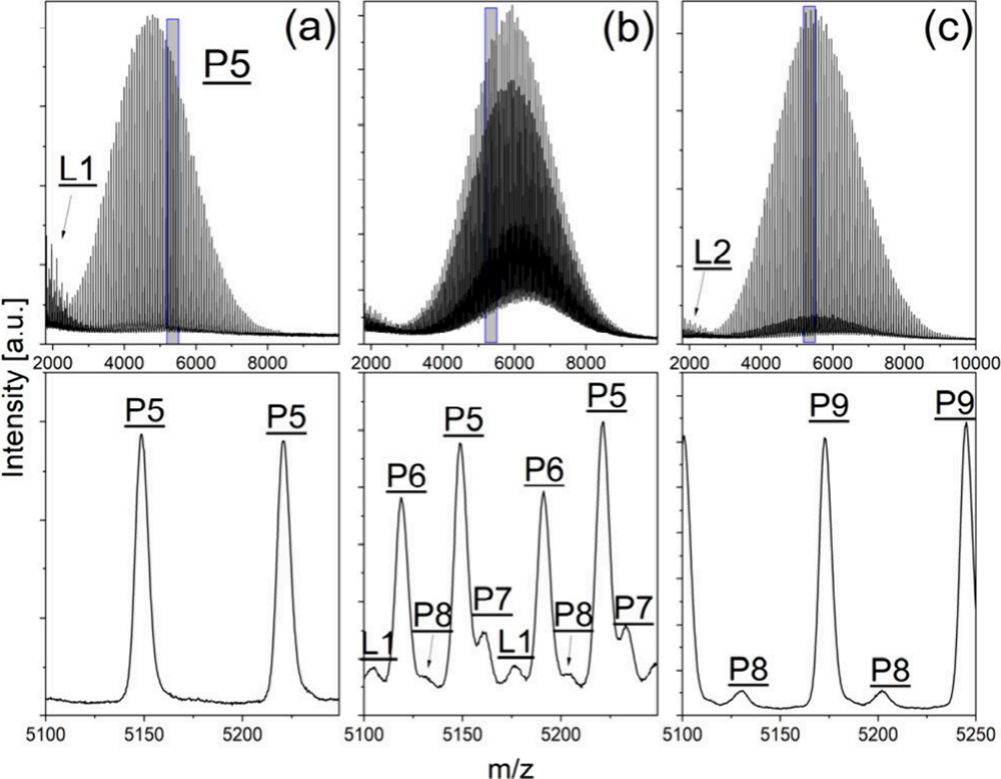
MALDI mass spectra of the original four-arm PLA (**P5**, a) and of two functionalization experiments (b, PENT–PLA-Ac_1; and c, PENT–PLA-Ac_2). Gray regions are expanded below, showing the change of peak pattern indicating different degrees of functionalization.

The second part of our investigation involved the functionalization of the four-arm PLA stars. The MALDI-TOF mass spectra of the original four-arm PLA star and of two experiments resulting in different degrees of functionalization (PENT–PLA-Ac_1 and PENT–PLA-Ac_2) are shown in Figure 4. In particular, the enlargement of the mass spectra of PENT–PLA-Ac_1 (Figure 4b) demonstrates the complexity of the sample and the difficulties in assigning structures clearly. From this spectrum, it can be concluded that product **P9**, representing the fully functionalized four-arm star, was not formed, whereas experiment PENT–PLA-Ac_2 (Figure 4c) showed almost complete functionalization.

**Figure 5:**
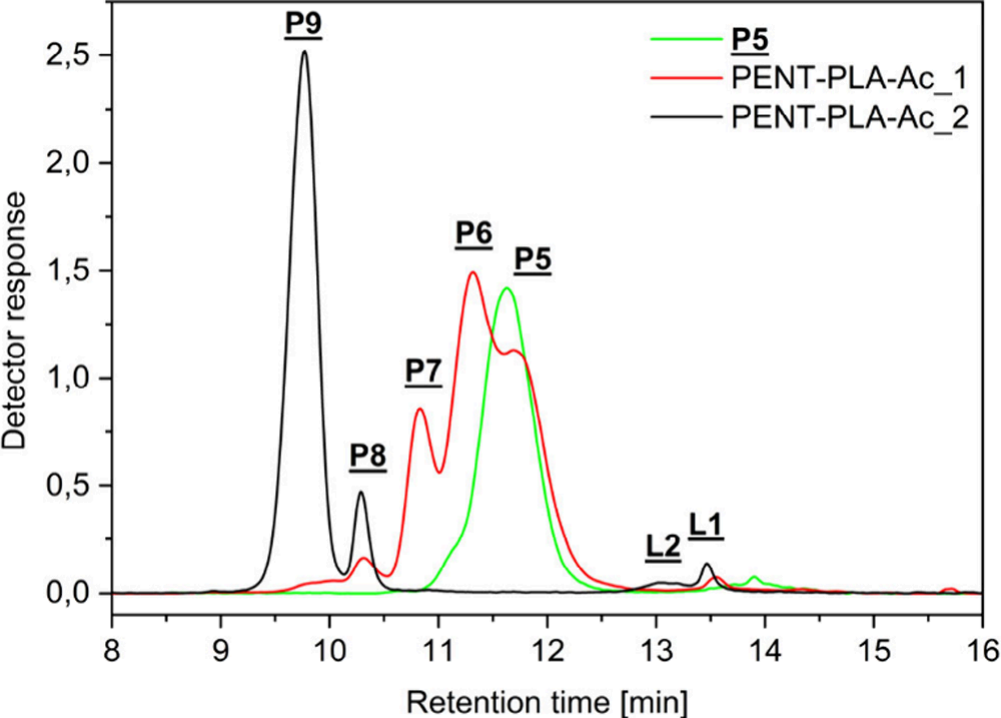
Overlay of GELAC elution curves of the original four-arm PENT–PLA (**P5**) and after functionalization (PENT–PLA-Ac_1 and PENT–PLA-Ac_2). The region between 13 and 14.5 min shows lower mass linear PLA (**L1**, **L2**).

Similar to the three-arm PLA stars, GELAC analyses were performed with these products (see Figure 5).

These chromatograms clearly confirmed the MALDI data and showed that the sample PENT–PLA-Ac_1 consisted of PLA stars with different degrees of acetylation. However, in contrast to the MALDI-TOF mass spectra, where no peaks of **P9** could be detected, traces of it could be found in the chromatogram, indicated by a tiny shoulder at 9.8 min. In contrast, the chromatogram of the PENT–PLA-Ac_2 experiment showed almost complete functionalization (**P9**). Only a minor amount of **P8**, indicated by the peak at 10.2 min, remained incompletely functionalized.

**Figure 6:**
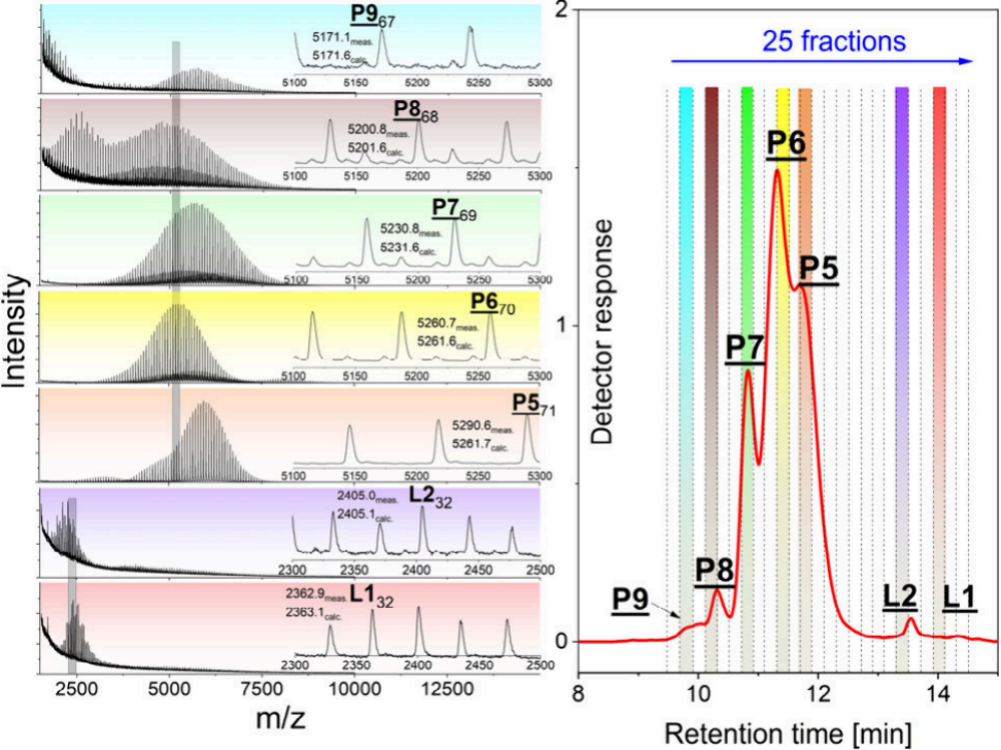
MALDI-TOF mass spectra of characteristic fractions from the GELAC run of sample PENT–PLA-Ac_1 showing various end group functionalities (for assignment, see Scheme 2).

These two samples (PENT–PLA-Ac_1 and PENT–PLA-Ac_2) were also used for chromatographic fractionation, followed by MALDI analysis of the fractions. In the first run, the incompletely functionalized sample PENT–PLA-Ac_1 was fractionated. The MALDI mass spectra of selected fractions are shown in Figure 6 and confirmed the supposed GELAC elution behavior. Again, a systematic shift of the peak series from **P9** to **P5** could be found.

Similar to the results of the three-arm PLA, additional peak series indicating the formation of potassium carboxylates were found for the linear side products. In a second fractionation experiment, sample PENT–PLA-Ac_2 was investigated. The results (shown in Figure S2, see Supporting Information) again confirmed almost complete end group modification. Only a minor part of **P8** with an incomplete modification of one arm was detected.

**Figure 7:**
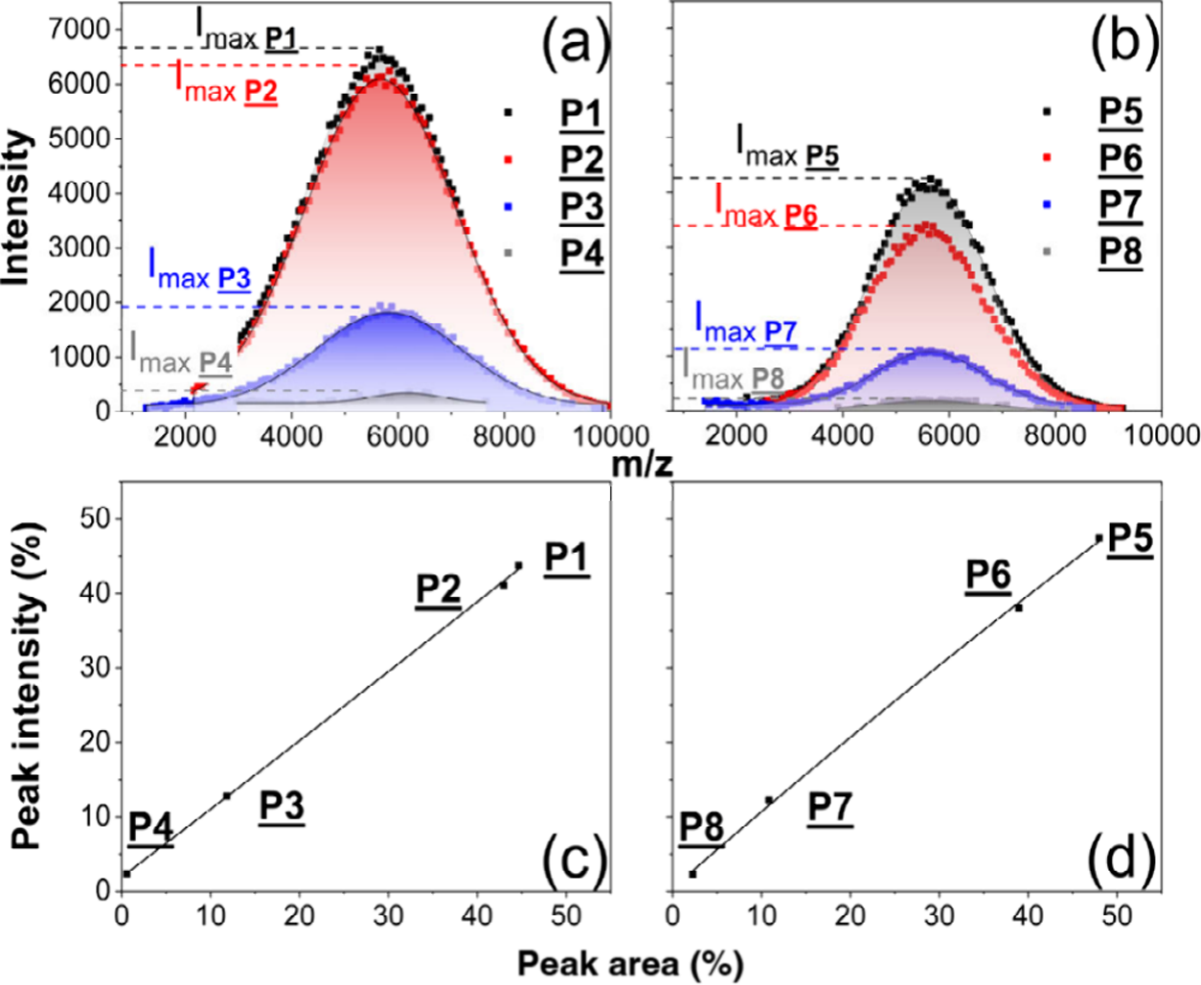
Comparison of peak fitting methods: area of complete peak distribution vs intensity of peaks at a maximum of distribution of TMP-PLA-Ac_2 (a) and PENT–PLA-Ac_1 (b) and correlation curve between both methods (c + d).

The next step was to compare the results of the two analytical techniques. While the software used for the chromatography allowed easy peak fitting, the calculation of the MS data was more difficult. Therefore, the first test was to compare MALDI peak intensities with MALDI peak areas. This was done by comparing the single peak intensity at the maximum of each functionalization product with data derived from the total area under the entire peak distribution, as shown exemplarily for the experiments TMP-PLA-Ac_2 and PENT–PLA-Ac_1 in Figure 7a and b. Nearly identical results were obtained, shown by the linear intensity versus area plots (see Figure 7c and d), demonstrating that the simpler method of using intensities at the peak maxima provided sufficient accuracy.

**Figure 8:**
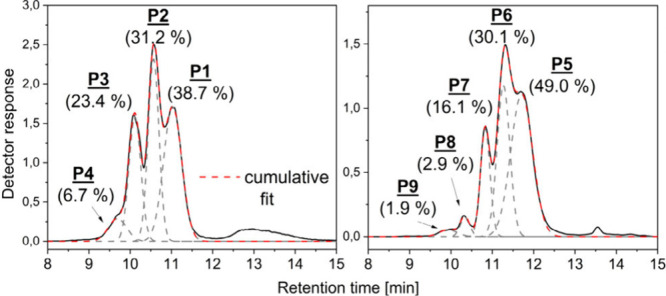
Peak fitting of chromatographic run (coefficient of determination (COD R2 > 0.99) and calculated peak area of a three-arm TMP-PLA-Ac_2 (left) and a four-arm PENT–PLA-Ac_1 (right) PLA star.

**Figure 9:**
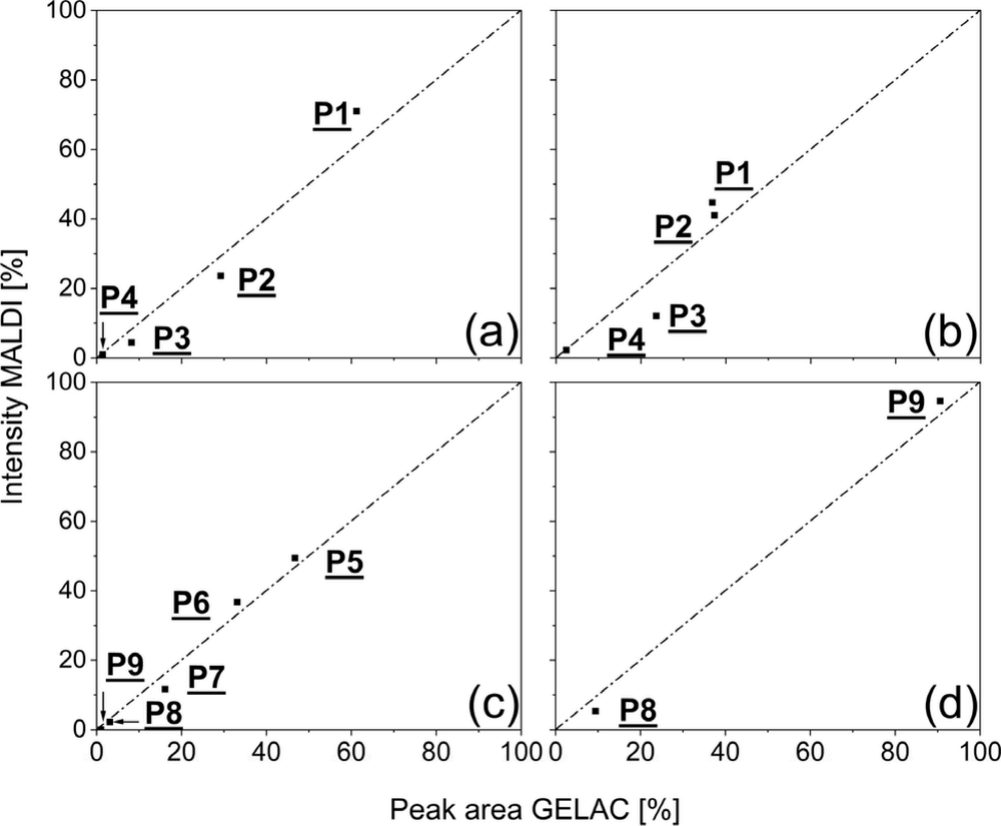
Comparison of GELAC areas and MALDI-TOF MS intensities of three- and four-arm PLA stars with different degree of acetylation, TMP-PLA-Ac_1 (a), TMP-PLA-Ac_2 (b), PENT–PLA-Ac_1 (c), and PENT–PLA-Ac_2 (d).

Figure 8 (and Figures S3–S5) shows two representative chromatograms along with the peak fitting data (Gaussian fit function, Origin 2023 Pro). These data were compared with the results extracted from the intensity information on the MALDI MS spectra (see Table S2, Supporting Information) and plotted in Figure 9. As shown in relation to an ideal 1:1 behavior (straight dotted lines in Figure 9), a good correlation between MALDI and GELAC was observed.

While the results obtained for the PENT–PLA-Ac experiments show an almost 1:1 correlation, the correlation of the intensities observed in the TMP-PLA-Ac experiments (Figure 9a + b) showed a larger deviation from an ideal 1:1 ratio. The largest deviation was observed for the fraction **P3** (Figure 9b), which showed the lowest abundance in the MALDI spectra and is also not completely separated from peaks of **P1** (see expended regions of Figure 1b,c). In addition to the problem of insufficient MALDI peak resolution, there is also the question of whether this discrepancy is due to the use of an incorrect peak-fitting algorithm for the relatively complex chromatographic runs or due to a different response of the ELSD detector used. Therefore, another peak fitting function (Gauss-Lorenz, Origin 2023 PRO) was tested first and compared with the previously used Gauss-fit function.

Although both fits gave excellent *R*^2^ and *C*^2^ values, areas calculated were completely different (see Figure S6). A decision as to whether the fit function produced (more) reliable data could not be made at this time.

Another possibility would have been to tune the chromatographic resolution to obtain better resolved peaks in GELAC. However, this was not done, as it would increase the range in which the composition of the mobile phase changes, leading to a significant alteration of the detector response.

Therefore, some kind of calibration was necessary, which could only be done with samples that are fully baseline-separated from each other. For this experiment, equimolar mixtures of **P1** (TMP–OH) and fully acetylated **P4** (from the TMP-PLA-Ac_3 experiment), respectively **P5** (PENT–OH) and its fully acetylated star **P9** (from the PENT–PLA-Ac_2 experiment), were prepared. As mentioned above and shown in Figure S2, sample **P9** contained a small amount of **P8** (approximately 5–9% depending on the analytical technique chosen), which had to be considered for peak evaluation by MALDI TOF MS and GELAC. The observed spectra and chromatograms are shown in Figures S7 (MALDI) and S8 (GELAC). Figure S7 also shows that acetylation of the OH end groups caused only a slight but noticeable increase in the peak intensities in the MALDI-TOF mass spectra.

**Figure 10:**
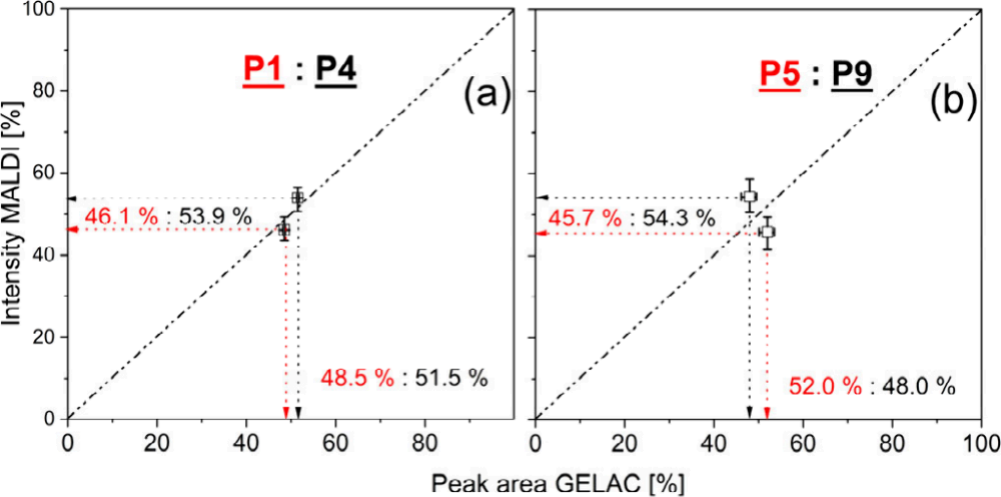
Comparison of GELAC peak areas (least five repetitions) and MALDI-TOF MS intensities (eight repetitions) of equimolar blends of neat and completely acetylated PLA stars: (a) three-arm (**P1** + **P4**) and (b) four-arm (**P5** + **P9**).

A comparison of these two results is made in Figure 10 and shows that there is a close correlation between the chromatographic and mass spectrometric data of the three-arm stars, whereas in the case of the four-arm stars these data differed by almost 7%. Obviously, the largest contribution to this variation came from the MALDI data, as shown by the error bars in Figure 10 (± 4.3%), whereas the reproducibility of the chromatographic evaluation, verified by five to six replicates, was much better (± 1.8%). This is not surprising, as recent investigations have shown that there are certainly more issues to consider in MALDI-TOF mass spectrometry, presumably related to sample preparation and sample spot homogeneity.^17^ However, this is an ongoing investigation and will be the subject of a future publication.

## Conclusions

The results obtained show that MALDI-TOF MS in combination with chromatography can be used to estimate the degree of functionalization of acetylation of multiarm PLAs. However, it should be noted that especially in complex mass spectra there is always a chance to miss species present in comparatively low abundance (e.g., about 2% of **P9** in PENT–PLA-Ac_1).

The stepwise substitution of hydroxyl by acetyl end groups caused only a slight increase in the signal intensity in MALDI-TOF mass spectrometry, but this is within the range of uncertainty, which may well be caused by the inhomogeneities of the MALDI sample preparation. Thus, no significant effect of functionalization on the spectral intensity was found. Two conclusions can be drawn. Either both end groups cause similar ionization probabilities in the MALDI process or they are not involved in ionization. A clear decision can be made only by the introduction of yet another end group structure.

Our results also show that the evaluation of chromatographic data by peak fitting of incompletely separated curves is not straightforward. On the other hand, the good correlation of MALDI and GELAC data for fully baselined stars can be used to evaluate the suitability of the fitting procedure for poorly separated samples in chromatography.

## Supporting Information Available

(Table S1) Molar masses of synthesized star-shaped polylactides; (Table S2) relative intensities of species with different degree of acetylation; (Figure S1) MALDI-TOF mass spectra of characteristic fractions from the GELAC run of sample TMP-PLA-Ac_3; (Figure S2) MALDI-TOF mass spectra of characteristic fractions from the GELAC run of sample PENT–PLA-Ac_2; (Figure S3) peak fitting of the chromatographic runs of a three-arm PLA star (TMP-PLA-Ac_1); (Figure S4) peak fitting of the chromatographic runs of a three-arm PLA star (TMP-PLA-Ac_3); (Figure S5) peak fitting of the chromatographic runs of a four-arm PLA star (PENT–PLA-Ac_2); (Figure S6) comparison of peak fitting using two different fitting functions, (a + c) Gauss-Lorenz, (b + d) Gauss; (Figure S7) MALDI TOF mass spectra of a 1:1 (molar) mixture of **P1** and **P4** (a) and **P5** and **P9** (b); (Figure S8) GELAC chromatograms of a 1:1 (molar) mixture of **P1** and **P4** (left) and **P5** and **P9** (right) (PDF)
