# Supplementary material for: Quantitative MALDI-TOF Mass Spectrometry of Star-Shaped Polylactides Based on Chromatographic Hyphenation
Source: J Am Soc Mass Spectrom. 2025 Jan 30;36(3):613–21. doi: 10.1021/jasms.4c00491 (PMC11887435; doi:10.1021/jasms.4c00491)
Supplement: Supplementary file 1 — js4c00491_si_001.pdf [file js4c00491_si_001.pdf]

Supplemental Part

to

Quantitative MALDI-TOF mass spectrometry of star-shape polylactides based on chromatographic hyphenation

Jana Falkenhagen, Mete-Sungur Dalgic and Steffen M. Weidner*

Federal Institute for Materials Research and Testing (BAM), Richard-Willstätter-Strasse 11, D-12489 Berlin, Germany

*author for correspondence: steffen.weidner@bam.de

Table S1 Molar masses of synthesized star-shaped polylactides calculated by SEC with different calibrants and MALDI-TOF MS

Sample	SEC						MALDI-TOF MS [a)]
	PS standards calibration [kg mol^{-1}]			PLA standards calibration [kg mol^{-1}]			[m/z]
	Mw	Mn	Mp	Mw	Mn	Mp	Mp
**P1**	12.1	10.0	13.0	4.9	4.3	5.1	4.7
TMP-PLA-Ac _1	11.4	8.9	12.5	4.7	4.0	4.9	4.4
TMP-PLA-Ac _2	11.3	8.9	12.4	4.6	3.9	4.9	5.3
TMP-PLA-Ac _3	12.3	10.0	13.2	5.0	4.3	5.2	5.3
**S5**	11.4	9.3	12.2	4.6	4.1	4.8	4.8
PENT-PLA-Ac_1	12.1	10.8	12.8	4.9	4.4	5.0	5.5
PENT-PLA-Ac_2	11.9	10.0	12.7	4.8	4.3	5.0	5.4

a – average of 4 measurements

Table S2 Relative intensities of species with different degree of acetylation taken from peak fitting of chromatographic runs (Gauss fit) and from highest peak intensities of MALDI spectra

	TMP-PLA-Ac_1		TMP-PLA-Ac_2		TMP-PLA-Ac_3		PENT-PLA-Ac_1		PENT-PLA-Ac_2	
	GELAC	MALDI	GELAC	MALDI	GELAC	MALDI	GELAC	MALDI	GELAC	MALDI
**P1**	61.2	71.0	37.8	44.7	0	0				
**P2**	29.2	23.6	34.3	41.0	0	0				
**P3**	8.2	4.4	23.5	12.1	0	0				
**P4**	1.4	1.0	4.4	2.2	100	100				
**P5**							46.7	49.4		
**P6**							33.1	36.7		
**P7**							16.1	11.7		
**P8**							3.1	2.2	9.4	5.4
**P9**							1.0	0.0	90.6	94.6

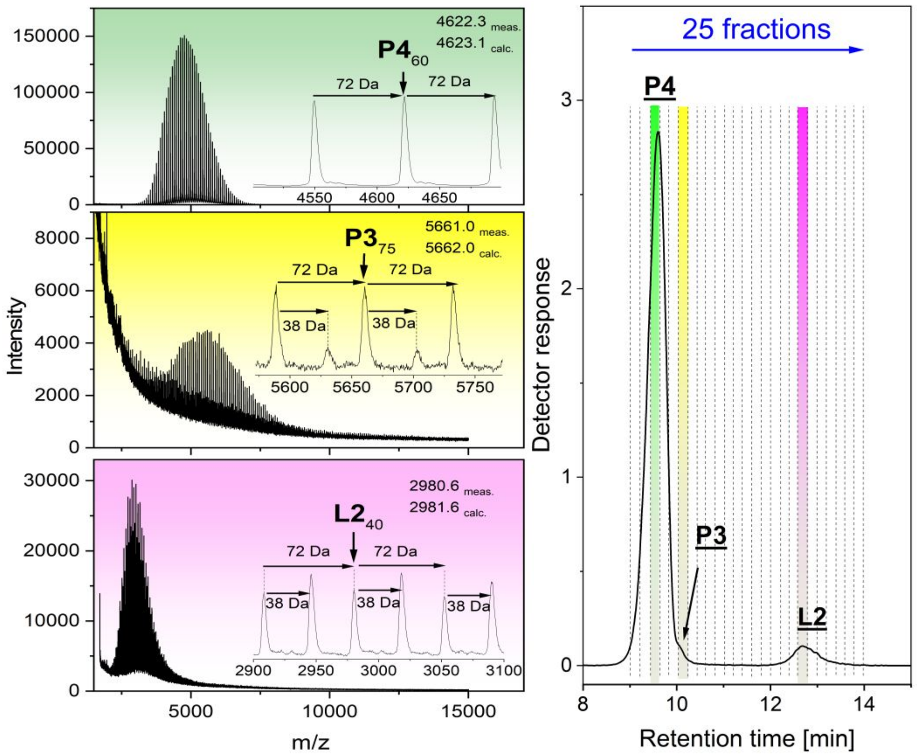

Figure S1 MALDI-TOF mass spectra of characteristic fractions from the GELAC run of sample TMP-PLA-Ac_3 showing three different end group functionalities (for assignment see Figure 1)

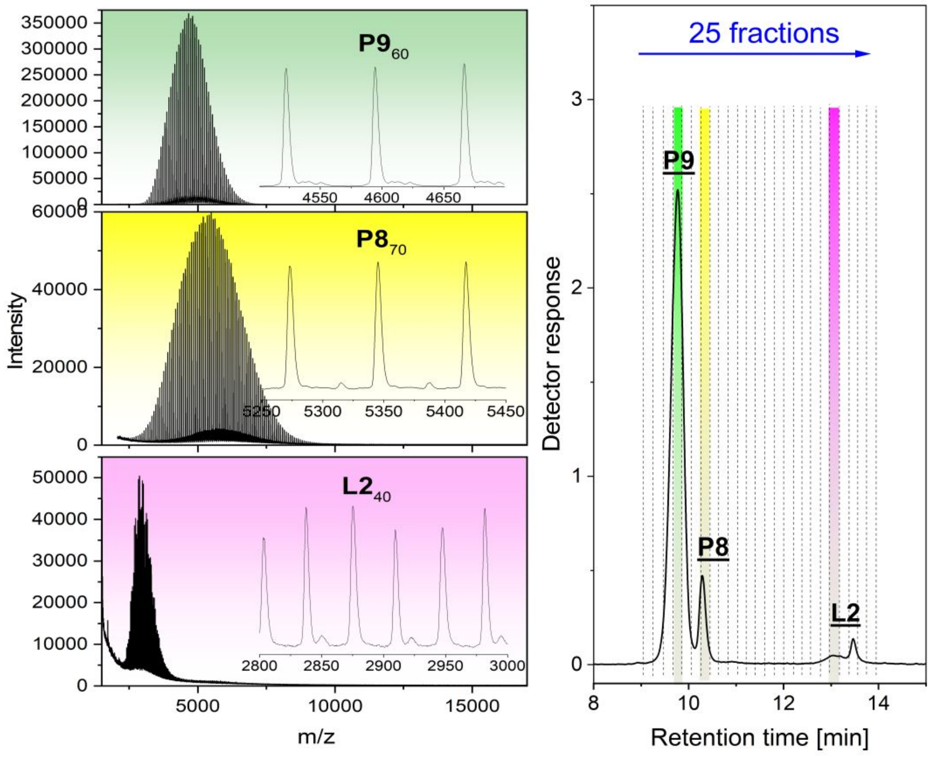

Figure S2 MALDI-TOF mass spectra of characteristic fractions from the GELAC run of sample PENT-PLA-Ac_2 showing three different end group functionalities (for assignment see Figure 4).

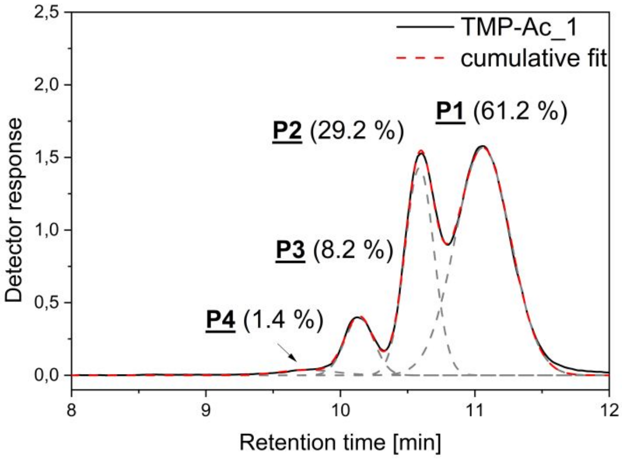

Figure S3 Peak fitting of the chromatographic runs (coefficient of determination (COD R^2) > 0.99) and calculated peak area of a 3-arm PLA star (TMP-PLA-Ac_1)

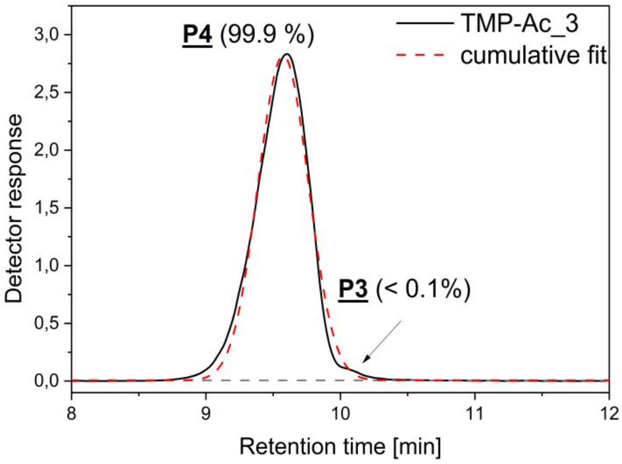

Figure S4 Peak fitting of the chromatographic runs (coefficient of determination (COD R^2) > 0.99) and calculated peak area of a 3-arm PLA star (TMP-PLA-Ac_3)

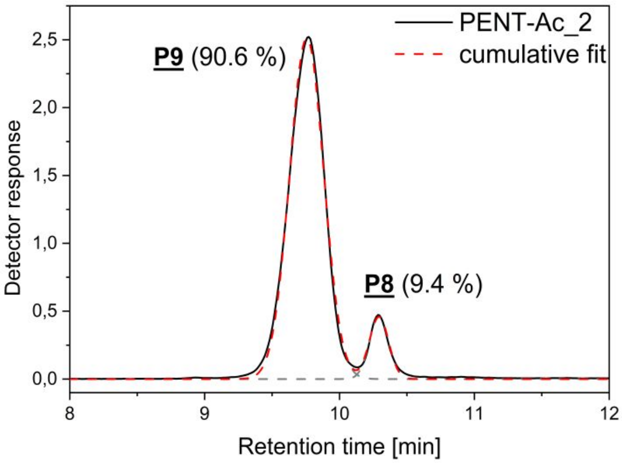

Figure S5 Peak fitting of the chromatographic runs (coefficient of determination (COD R^2) > 0.99) and calculated peak area of a 4-arm PLA star (PENT-PLA-Ac_2)

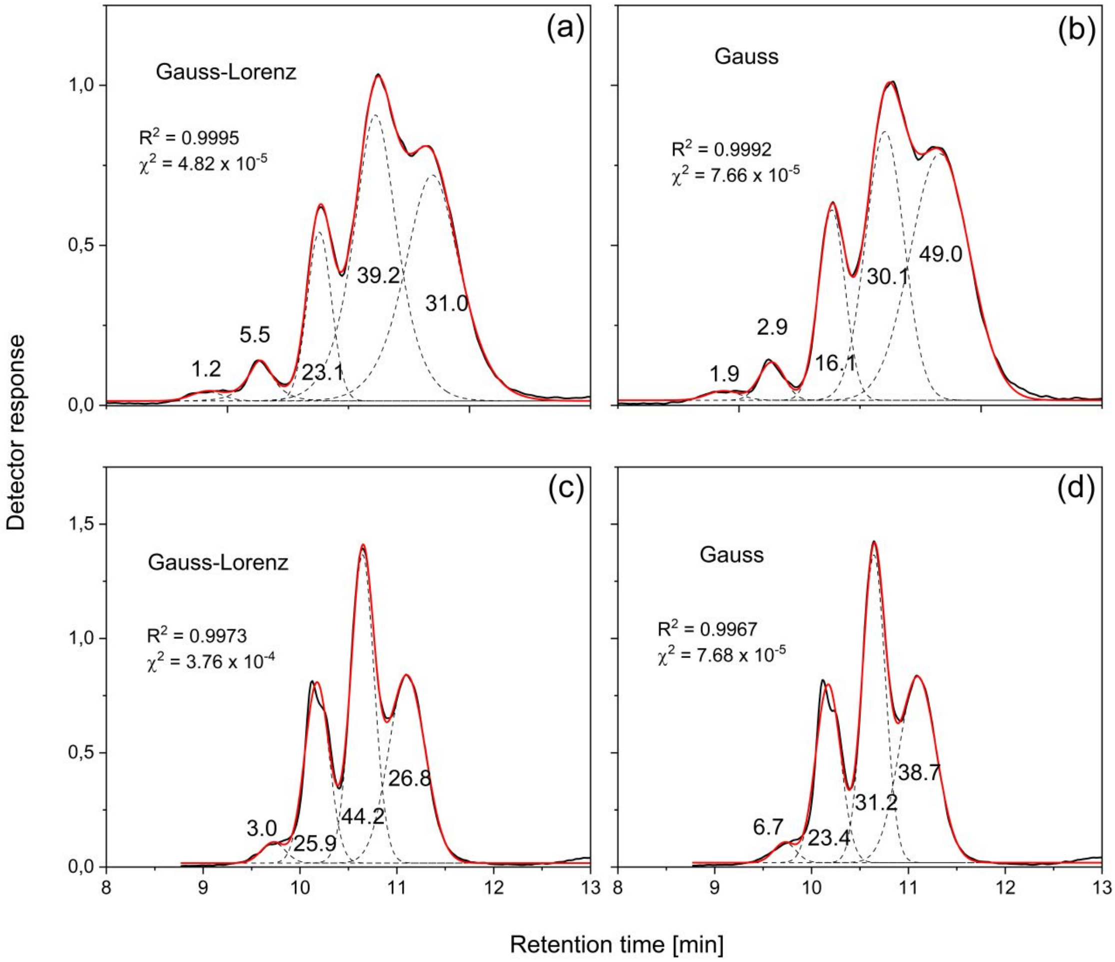

Figure S6 Comparison of peak fitting of GELAC elugrams of PENT-PLA-Ac_1 (top) and TMP-PLA-Ac_2 (bottom) using two different fitting functions, (a + c) Gauss-Lorenz, (b + d) Gauss

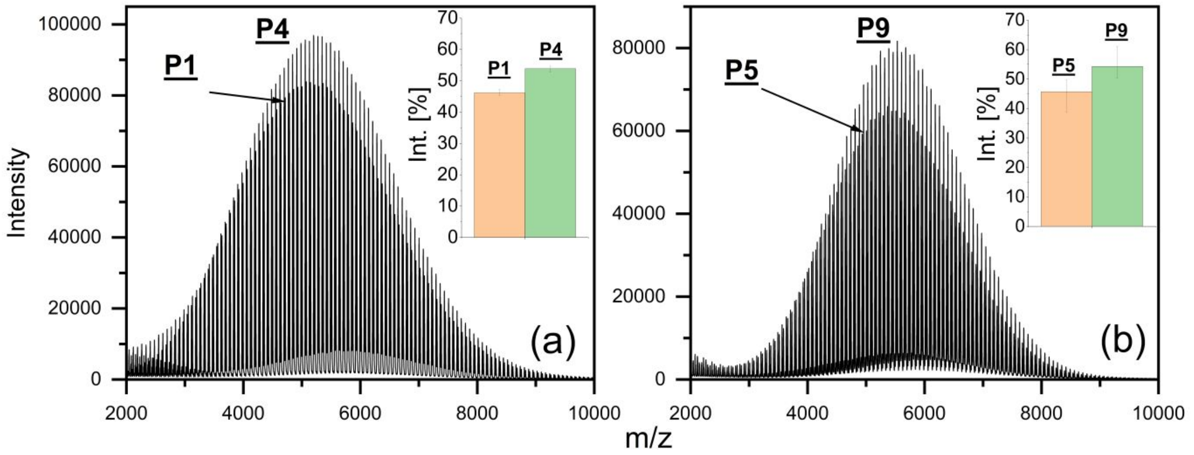

Figure S7 MALDI TOF Mass spectra of a 1:1 (molar) mixture of **<u>P1</u>** and **<u>P4</u>** (a) and **<u>P5</u>** and **<u>P9</u>** (b) and extracted intensity ratios based on peak maximum intensities (insert)

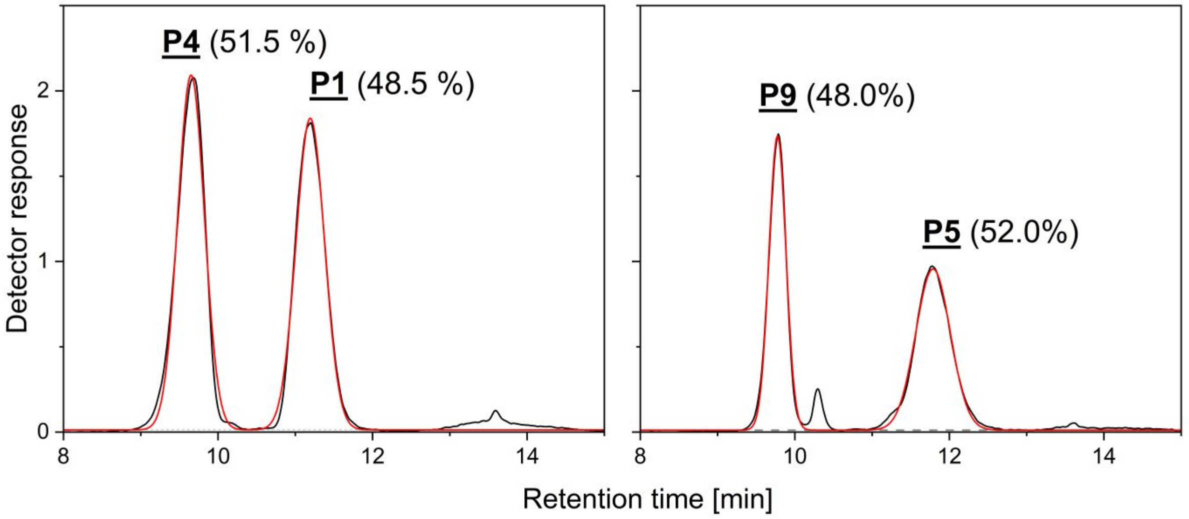

Figure S8 GELAC chromatograms of a 1:1 (molar) mixture of **<u>P1</u>** and **<u>P4</u>** (left) and **<u>P5</u>** and **<u>P9</u>** (right) and ratio of peak area
